# Crystal structure and Hirshfield analysis of the 4-(di­methyl­amino)­pyridine adduct of 4-meth­oxy­phenyl­borane

**DOI:** 10.1107/S2056989017015171

**Published:** 2017-10-20

**Authors:** Jesse Shooter, Caleb J. Allen, Colby W. K. Tinsley, Lev N. Zakharov, Eric R. Abbey

**Affiliations:** a Department of Chemistry, Biochemistry, and Physics, Eastern Washington, University, Cheney, WA 99004, USA; b Department of Chemistry and Biochemistry, CAMCOR, University of Oregon, Eugene, OR 97403, USA

**Keywords:** crystal structure, hydrogen bonding, zwitterions, Hirshfield analysis

## Abstract

The title compound [systematic name: 4-(di­methyl­amino)­pyridine–4-meth­oxy­phenyl­borane (1/1)], C_14_H_19_BN_2_O, contains two independent mol­ecules in the asymmetric unit. Both molecules exhibit coplanar, mostly *sp* ^2^-hybridized meth­oxy and di­methyl­amino substituents on their respective aromatic rings, consistent with π-donation into the aromatic systems. The B—H groups exhibit an intra­molecular close contact with a C—H group of the pyridine ring, which may be evidence of electrostatic attraction between the hydridic B—H and the electropositive aromatic C—H. There appears to be weak C—H⋯π(arene) inter­actions between two of the H atoms of an amino­methyl group and the meth­oxy-substituted benzene ring of the other independent mol­ecule, and another C—H⋯π (arene) inter­action between one of the pyridine ring H atoms and the same benzene ring.

## Abstract 2

The asymmetric unit contains two independent mol­ecules, which exhibit coplanar, mostly *sp* ^2^-hybridized meth­oxy and di­methyl­amino substituents on their respective aromatic rings, consistent with π-donation into the aromatic systems. The B—H groups exhibit an intra­molecular close contact with a C—H group of the pyridine ring, which may be evidence of electrostatic attraction between the hydridic B—H and the electropositive aromatic C—H.

## Chemical context   

Monoorganoboranes (*R*BH_2_) have been the focus of chemical research for over fifty years, most notably for their use in the indispensable hydro­boration reaction, which permits reduction of olefins, carbonyl compounds and others (Brown & Krishnamurthy, 1979 ▸; Crudden & Edwards, 2003 ▸.) Such boranes are often isolated as their Lewis base adducts, in which the base donates a lone pair into the vacant *p* orbital of the *sp^2^* borane. Among the most common class of Lewis bases for the formation of borane adducts are amines. Amine boranes are widely used as hydro­boration reagents (Clay & Vedejs, 2005 ▸), precursors for borenium cation synthesis (De Vries *et al.*, 2012 ▸), frustrated Lewis pairs (Stephan, 2015 ▸), and have been investigated as hydrogen-storage materials (Campbell *et al.*, 2010 ▸). We have synthesized the zwitterionic title compound by hydride removal from sodium 4-meth­oxy­phenyl­borohydride with chloro­tri­methyl­silane in the presence of 4-di­methyl­amino­pyridine. This compound is slightly unusual, as examples of monoorganoboranes with hetero­atoms on the organic substitituent are limited.

## Structural commentary   

**Figure 1:**
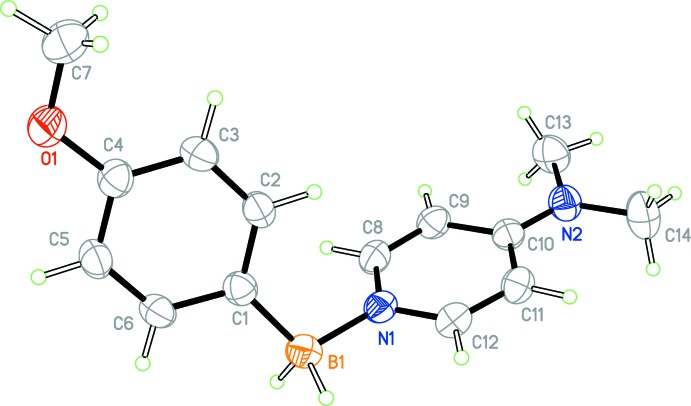
The mol­ecular structure of one of the independent mol­ecules of the title compound with displacement ellipsoids drawn at the 50% probability level.

**Figure 3:**
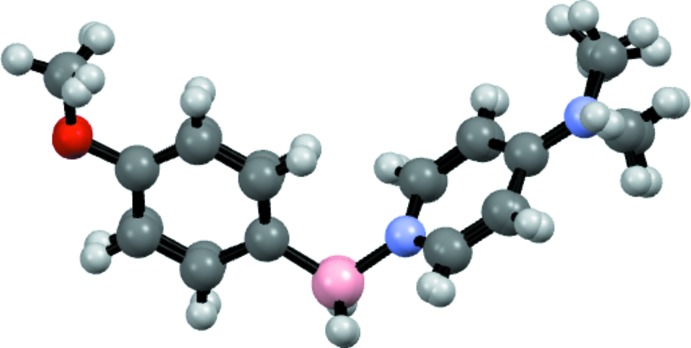
An overlay of the two independent mol­ecules.

The asymmetric unit contains two independent mol­ecules (Figs. 1 ▸ and 2 ▸) with only slightly different geometric features (Fig. 3 ▸). In both mol­ecules, the boron atom appears to be *sp^3^* hybridized [C1—B1—N1 = 110.8 (1) and C1′—B1′—N1′ = 111.0 (1)°] . The B1—C1 and B1′—C1′ distances [1.608 (2) and 1.611 (2) Å, respectively] are consistent with a formal C—B single bond. The oxygen atom of both meth­oxy groups appears to be mostly *sp^2^* hybridized, [C7—O1—C4 = 117.3 (1) and C7′—O1′—C4′ = 117.4 (1)°] and is close to coplanar with the phenyl ring [torsion angles C7—O1—C4—C3 = −7.4 (2) and C7′—O1′—C4′—C3′ = −7.1 (2)°], consistent with π-donation into the phenyl ring.
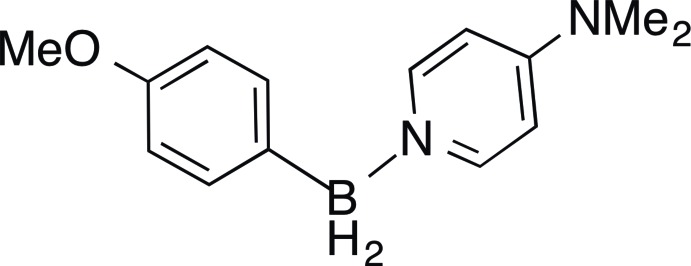


The geometries of the 4-(dimethylamino)pyridine (DMAP) fragment of both mol­ecules is similar to other structures of DMAP–borane adducts. The nitro­gen atom of the di­methyl­amino fragment appears to be *sp^2^* hybridized [torsion angles C13—N2—C10 = 121.0 (1)° and C13′—N2′—C10′ = 122.2 (1)°] and is close to coplanar [torsion angles C13—N2—C10—C11 = 2.4 (2) and C13′—N2′—C10′—C11′ = 3.4 (1)°] consistent with π-donation into the pyridine ring.

The B1—N1 and B1′—N1′ distances [1.597 (2) and 1.595 (2) Å, respectively] are consistent with formal N—B single bonds, and are within the range observed for other DMAP–borane adducts (see *Database survey*). Inter­estingly, the B—H atoms exhibit intra­molecular close contacts with the C—H atoms of the pyridine ring [H12⋯H2*B* = 2.26 (3) and H12′⋯H2*B*′ = 2.27 (3) Å] and are close to coplanar [torsion angles H2*B*—B1—N1—C12 = 4(1) and H2*B*—B1—N1—C12 = 16 (1)°], which may be evidence of electrostatic inter­actions between the hydridic B—H atoms and electropositive aromatic C—H atoms, and is observed in other DMAP–borane adducts (see *Database Survey*). The planes of the pyridine rings and the benzene rings are almost normal to one another [the dihedral angle between the C1–C6 and C8–C12/N1 rings is 73.14 (7)° and that between the C1′–C6′ and C8′–C12′/N1′ rings is 74.15 (7)°]. Perhaps the most significant difference between the two mol­ecules is the 9.0° difference in the torsion angle about the B—N bond [C1—B1—N1—C8 = −63.9 (2) while C1′—B1′—N1′—C8′ = −72.9 (2)°] (Fig. 3 ▸).

## Supra­molecular features   

**Figure 4:**
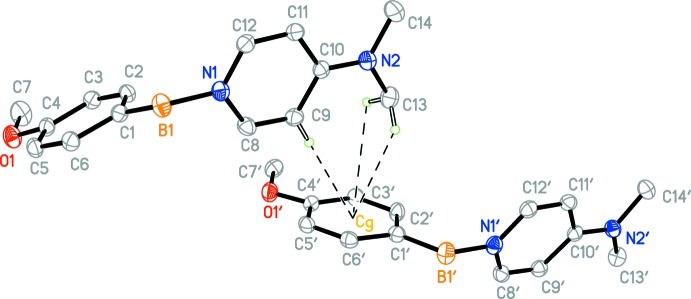
Weak C—H⋯π (arene) inter­actions between the two independent mol­ecules in the unit cell shown as dashed lines. *Cg* is the centroid of the C1′–C6′ benzene ring. Only H atoms involved in the inter­actions are shown.

The mol­ecules within the asymmetric unit exhibit weak C—H⋯π (arene) inter­actions between two of the hydrogen atoms of the amino­methyl group and the meth­oxy­phenyl group of a neighboring mol­ecule (see Table 1 ▸) as well as a C—H⋯π(arene) inter­action between one of the pyridine hydrogen atoms and the same meth­oxy­phenyl ring (Fig. 4 ▸).

## Hirshfield analysis   

**Figure 5:**
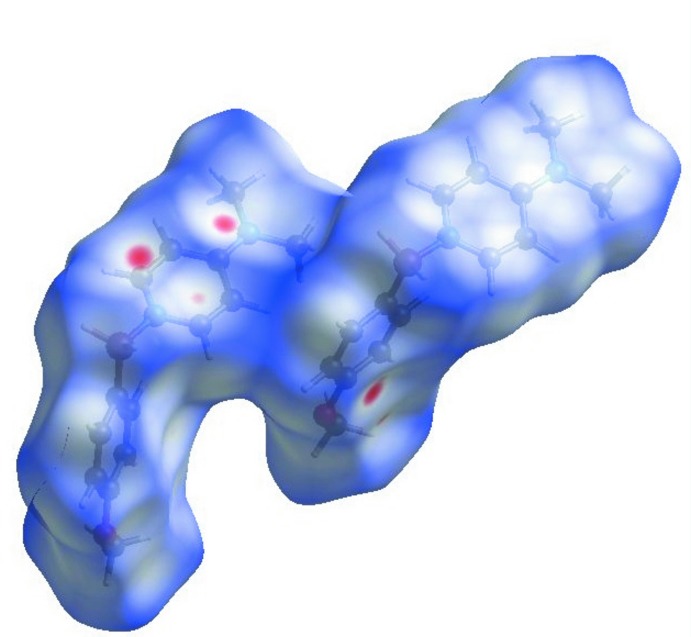
Hirshfield surface mapped over *d*_norm_. Red areas highlight inter­molecular contacts shorter than the sum of the van der Waals radii.

The weak inter­molecular inter­actions of the title compound were explored by Hirshfield analysis. Hirshfield surfaces were generated using *Crystal Explorer 3.1* (McKinnon *et al.*, 2007 ▸; Spackman & Jayatilaka, 2009 ▸). The space within a crystal is partitioned so that the ratio of promolecule to procrystal is equal to 0.5, generating continuous surfaces that permit the visualization of weak inter­actions. The *d*_norm_ values illustrate whether the inter­molecular contact is shorter or longer than the van der Waals radii. Red areas of the Hirshfield surface indicate negative *d*_norm_ values contacts closer than the van der Waals radii. This analysis lends further support to the weak C—H^⋯^π (arene) inter­actions described in the previous section (Fig. 5 ▸.)

## Database survey   

A search of the Cambridge Structural Database (Version 5.37, update February 2017; Groom *et al.*, 2016 ▸) for DMAP–borane adducts yielded only two structures: VOGJEI (Chu, *et al.*, 2014 ▸) and JUDQAA (Lesley *et al.*, 1998 ▸). A search for phenyl-based monoorganoborane–amine adducts (Ph–BH_2_–N*R*_3_) yielded four structures: UTOZEJ (Hubner *et al.*, 2012 ▸), BEXQOM (Ménard & Stephan, 2013 ▸), EPOYAK (Franz *et al.*, 2011 ▸), and GEBNAE (Jacobs *et al.*, 2012 ▸). In all four of these structures, the B—N bonds are approximately perpendicular to the plane of the arene rings. In all six cases, the boron atom is tetra­hedral and displays structural features consistent with *sp^3^* hybridization. Additionally, the C—B and B—N bonds are all within the range for formal C—B and C—N single bonds.

## Synthesis and crystallization   

In a nitro­gen-filled glove box, sodium 4-meth­oxy­phenyl­borohydride (97mg, 0.67 mmol) and 4-di­methyl­amino­pyridine (82 mg, 0.67 mmol) were combined in a 20 mL vial containing a stir bar and dissolved in anhydrous THF (4 mL). The solution was cooled to 247 K in the freezer and chloro­tri­methyl­silane (73 mg, 0.67 mmol) was added dropwise *via* syringe. The reaction was allowed to come to 295 K and was stirred for 1 h. The solvent was then removed *in vacuo* and the residue was washed with anhydrous diethyl ether (4 mL), followed by extraction with anhydrous di­chloro­methane (4 mL). The extract was filtered through a 0.45 µm PTFE syringe filter. The solvent was again removed *in vacuo* to afford a white solid (51 mg, 37%). Crystals suitable for X-ray diffraction were grown by diffusion of pentane into a concentrated solution of the title compound in anhydrous di­chloro­methane.

^1^H NMR (500 MHz, CDCl_3_) δ (ppm): 8.12 (*d*, 2H, *J =* 7 Hz), 7.23 (*d*, 2 H, *J* = 8 Hz), 6.80 (*d*, 2H, *J* = 8.5 Hz), 6.52 (*d*, 2H, *J* = 8 Hz), 3.78 (*s*, 3H), 3.11 (*s*, 6H). ^13^C NMR (126 MHz, CDCl_3_) δ (ppm): 157.3, 154.9, 146.7, 145.0 (*br s*), 134.5, 122.9, 106.5, 55.0, 39.5. ^11^B NMR (160 MHz, CDCl_3_) δ (ppm): −5.0 (*br*, *s*). FTIR (ATR, cm^−1^): 3012, 2952, 2923, 2853, 2610, 2346, 2288, 2227, 1634, 1548, 1442, 1418, 1392, 1237, 1223, 1161, 1076, 1031, 811, 797, 548, 515.

## Refinement   

Crystal data, data collection and structure refinement details are summarized in Table 2 ▸. H atoms were refined in calculated positions (C—H = 0.95 Å with *U*_iso_(H) = 1.5*U*_eq_(C-methyl) and 1.2eq(C) for other H atoms. The B-bound H atoms were located in a difference-Fourier map and freely refined. Methyl H atoms were refined without restrictions on rotation around the C—C bonds, HFIX 138 in *SHELXL* (Sheldrick, 2015 ▸).

## Supplementary Material

Crystal structure: contains datablock(s) I. DOI: 10.1107/S2056989017015171/lh5853sup1.cif

Structure factors: contains datablock(s) I. DOI: 10.1107/S2056989017015171/lh5853Isup2.hkl

CCDC reference: 1580559

Additional supporting information:  crystallographic information; 3D view; checkCIF report

## Figures and Tables

**Figure 2:**
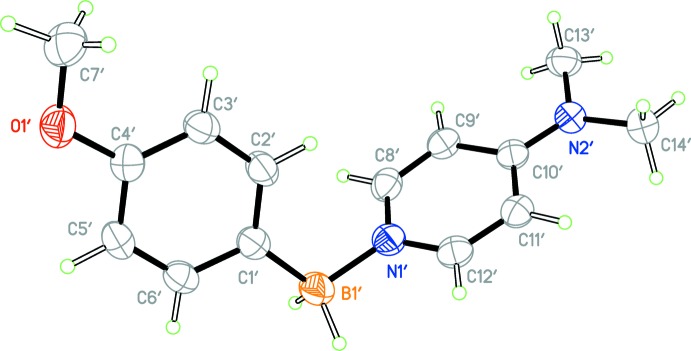
The mol­ecular structure of the other independent mol­ecule of the title compound with displacement ellipsoids drawn at the 50% probability level.

**Table 1:** Hydrogen-bond geometry (Å, °) *Cg* is the centroid of the C1′–C6′ ring.

*D*—H⋯*A*	*D*—H	H⋯*A*	*D*⋯*A*	*D*—H⋯*A*
C9—H9*A*⋯*Cg*	0.95	3.12	4.069 (2)	178
C13—H13*A*⋯*Cg*	0.97	3.12	3.662 (2)	112
C13—H13*C*⋯*Cg*	0.97	3.23	3.662 (2)	109

**Table 2:** Experimental details

Crystal data	
Chemical formula	C_14_H_19_BN_2_O
*M*_r_	242.12
Crystal system, space group	Orthorhombic, *P* *b* *c* *a*
Temperature (K)	173
*a*, *b*, *c* (Å)	12.3538 (6), 18.7727 (10), 23.4056 (12)
*V* (Å^3^)	5428.1 (5)
*Z*	16
Radiation type	Cu *K*α
μ (mm^−1^)	0.58
Crystal size (mm)	0.14 × 0.09 × 0.07

Data collection	
Diffractometer	Bruker APEXII CCD
Absorption correction	Multi-scan (*SADABS*; Bruker, 2008 ▸)
*T*_min_, *T*_max_	0.695, 0.753
No. of measured, independent and observed [*I* > 2σ(*I*)] reflections	46022, 4800, 3948
*R*_int_	0.063
(sin θ/λ)_max_ (Å^−1^)	0.595

Refinement	
*R*[*F* ^2^ > 2σ(*F* ^2^)], *wR*(*F* ^2^), *S*	0.047, 0.144, 1.08
No. of reflections	4800
No. of parameters	353
H-atom treatment	H atoms treated by a mixture of independent and constrained refinement
Δρ_max_, Δρ_min_ (e Å^−3^)	0.22, −0.22

Computer programs: *APEX2* and *SAINT* (Bruker, 2008 ▸), *SHELXS97* and *SHELXTL* (Sheldrick 2008 ▸), *SHELXL2014* (Sheldrick, 2015 ▸) and *Mercury* (Macrae *et al.*, 2008 ▸).

## supplementary crystallographic information

### Crystal data

**Table:**

C_14_H_19_BN_2_O	*D*_x_ = 1.185 Mg m^−^^3^
*M**_r_* = 242.12	Cu *K*α radiation, λ = 1.54178 Å
Orthorhombic, *P**b**c**a*	Cell parameters from 6122 reflections
*a* = 12.3538 (6) Å	θ = 3.8–66.5°
*b* = 18.7727 (10) Å	µ = 0.58 mm^−^^1^
*c* = 23.4056 (12) Å	*T* = 173 K
*V* = 5428.1 (5) Å^3^	Cut-block, colorless
*Z* = 16	0.14 × 0.09 × 0.07 mm
*F*(000) = 2080	

### Data collection

**Table:**

Bruker APEXII CCD diffractometer	3948 reflections with *I* > 2σ(*I*)
Radiation source: Incoatec IµS	*R*_int_ = 0.063
φ and ω scans	θ_max_ = 66.6°, θ_min_ = 3.8°
Absorption correction: multi-scan (SADABS; Bruker, 2008)	*h* = −14→14
*T*_min_ = 0.695, *T*_max_ = 0.753	*k* = −22→21
46022 measured reflections	*l* = −27→27
4800 independent reflections	

### Refinement

**Table:**

Refinement on *F*^2^	0 restraints
Least-squares matrix: full	Hydrogen site location: mixed
*R*[*F*^2^ > 2σ(*F*^2^)] = 0.047	H atoms treated by a mixture of independent and constrained refinement
*wR*(*F*^2^) = 0.144	*w* = 1/[σ^2^(*F*_o_^2^) + (0.0934*P*)^2^ + 0.2472*P*] where *P* = (*F*_o_^2^ + 2*F*_c_^2^)/3
*S* = 1.08	(Δ/σ)_max_ = 0.001
4800 reflections	Δρ_max_ = 0.22 e Å^−^^3^
353 parameters	Δρ_min_ = −0.22 e Å^−^^3^

### Special details

**Table:**

Geometry. All esds (except the esd in the dihedral angle between two l.s. planes) are estimated using the full covariance matrix. The cell esds are taken into account individually in the estimation of esds in distances, angles and torsion angles; correlations between esds in cell parameters are only used when they are defined by crystal symmetry. An approximate (isotropic) treatment of cell esds is used for estimating esds involving l.s. planes.

### Fractional atomic coordinates and isotropic or equivalent isotropic displacement parameters (Å^2^)

**Table:**

	*x*	*y*	*z*	*U*_iso_*/*U*_eq_	
B1	1.01984 (15)	0.31745 (11)	0.41477 (9)	0.0427 (4)	
O1	0.94372 (10)	0.19525 (7)	0.63937 (5)	0.0486 (3)	
N1	0.91495 (10)	0.31876 (7)	0.37491 (5)	0.0350 (3)	
N2	0.64797 (11)	0.31846 (7)	0.26602 (6)	0.0409 (3)	
C1	0.99199 (12)	0.28402 (8)	0.47629 (6)	0.0336 (3)	
C2	0.88854 (12)	0.27979 (8)	0.50007 (7)	0.0349 (3)	
H2A	0.8290	0.2968	0.4783	0.042*	
C3	0.86858 (12)	0.25193 (8)	0.55404 (7)	0.0367 (3)	
H3A	0.7969	0.2504	0.5686	0.044*	
C4	0.95416 (12)	0.22634 (8)	0.58646 (6)	0.0365 (3)	
C5	1.05847 (12)	0.23022 (9)	0.56465 (7)	0.0386 (4)	
H5A	1.1178	0.2135	0.5867	0.046*	
C6	1.07595 (12)	0.25838 (8)	0.51094 (7)	0.0364 (3)	
H6A	1.1480	0.2605	0.4969	0.044*	
C7	0.84022 (17)	0.19686 (13)	0.66503 (8)	0.0623 (5)	
H7A	0.8163 (7)	0.2472 (7)	0.6695 (7)	0.093*	
H7B	0.8437 (4)	0.1737 (9)	0.7032 (6)	0.093*	
H7C	0.7877 (8)	0.1708 (9)	0.6404 (5)	0.093*	
C8	0.86637 (13)	0.25774 (8)	0.35835 (7)	0.0384 (4)	
H8A	0.8952	0.2140	0.3720	0.046*	
C9	0.77855 (12)	0.25513 (8)	0.32330 (7)	0.0372 (3)	
H9A	0.7479	0.2104	0.3133	0.045*	
C10	0.73265 (12)	0.31892 (8)	0.30166 (6)	0.0331 (3)	
C11	0.78395 (13)	0.38231 (8)	0.31977 (7)	0.0365 (3)	
H11A	0.7572	0.4271	0.3072	0.044*	
C12	0.87174 (13)	0.37956 (8)	0.35519 (6)	0.0365 (3)	
H12A	0.9041	0.4232	0.3665	0.044*	
C13	0.59485 (15)	0.25190 (10)	0.25018 (8)	0.0502 (4)	
H13A	0.6465 (8)	0.2208 (5)	0.2318 (6)	0.075*	
H13B	0.5359 (11)	0.26183 (17)	0.2242 (6)	0.075*	
H13C	0.5669 (11)	0.2290 (5)	0.2841 (5)	0.075*	
C14	0.60151 (17)	0.38459 (11)	0.24483 (9)	0.0582 (5)	
H14A	0.5688 (13)	0.4106 (6)	0.2764 (5)	0.087*	
H14B	0.5465 (13)	0.37391 (18)	0.2163 (7)	0.087*	
H14C	0.6582 (9)	0.4135 (6)	0.2276 (7)	0.087*	
B1'	0.69503 (16)	0.06081 (12)	0.15670 (9)	0.0453 (5)	
O1'	0.60935 (10)	0.06495 (7)	0.40101 (5)	0.0465 (3)	
N1'	0.59005 (11)	0.04914 (7)	0.11816 (5)	0.0376 (3)	
N2'	0.31932 (10)	0.01508 (7)	0.01478 (6)	0.0381 (3)	
C1'	0.66395 (12)	0.06221 (8)	0.22354 (7)	0.0353 (3)	
C2'	0.55974 (13)	0.05774 (9)	0.24570 (7)	0.0390 (4)	
H2'A	0.5010	0.0533	0.2197	0.047*	
C3'	0.53692 (13)	0.05950 (9)	0.30416 (7)	0.0391 (4)	
H3'A	0.4642	0.0567	0.3172	0.047*	
C4'	0.62082 (13)	0.06528 (8)	0.34280 (7)	0.0355 (3)	
C5'	0.72647 (12)	0.07031 (8)	0.32248 (7)	0.0375 (4)	
H5'A	0.7850	0.0747	0.3486	0.045*	
C6'	0.74621 (12)	0.06892 (8)	0.26434 (7)	0.0365 (4)	
H6'A	0.8189	0.0727	0.2515	0.044*	
C7'	0.50222 (15)	0.06648 (10)	0.42327 (8)	0.0505 (4)	
H7'1	0.4633 (7)	0.1089 (7)	0.4080 (6)	0.076*	
H7'2	0.50509 (15)	0.0693 (8)	0.4658 (6)	0.076*	
H7'3	0.4632 (7)	0.0223 (7)	0.4117 (6)	0.076*	
C8'	0.54070 (13)	−0.01494 (9)	0.11568 (7)	0.0389 (4)	
H8'A	0.5701	−0.0528	0.1376	0.047*	
C9'	0.45100 (13)	−0.02840 (8)	0.08343 (7)	0.0370 (3)	
H9'A	0.4193	−0.0745	0.0839	0.044*	
C10'	0.40477 (12)	0.02603 (8)	0.04920 (6)	0.0335 (3)	
C11'	0.45656 (13)	0.09326 (8)	0.05317 (6)	0.0360 (3)	
H11B	0.4290	0.1326	0.0322	0.043*	
C12'	0.54561 (13)	0.10182 (8)	0.08691 (7)	0.0372 (3)	
H12B	0.5782	0.1476	0.0885	0.045*	
C13'	0.26225 (14)	−0.05283 (9)	0.01282 (8)	0.0450 (4)	
H13D	0.3125 (7)	−0.0908 (5)	0.0187 (6)	0.068*	
H13E	0.2285 (10)	−0.0583 (4)	−0.0236 (5)	0.068*	
H13F	0.2083 (10)	−0.0539 (3)	0.0421 (5)	0.068*	
C14'	0.26866 (14)	0.07399 (10)	−0.01557 (8)	0.0459 (4)	
H14D	0.2354 (11)	0.1068 (6)	0.0122 (4)	0.069*	
H14E	0.2125 (11)	0.0554 (2)	−0.0414 (5)	0.069*	
H14F	0.3238 (7)	0.0996 (6)	−0.0380 (5)	0.069*	
H1'B	0.7533 (16)	0.0147 (10)	0.1468 (8)	0.048 (5)*	
H1B	1.0840 (16)	0.2841 (11)	0.3930 (8)	0.051 (5)*	
H2'B	0.7314 (16)	0.1141 (10)	0.1398 (9)	0.052 (5)*	
H2B	1.0475 (17)	0.3752 (11)	0.4179 (9)	0.056 (6)*	

### Atomic displacement parameters (Å^2^)

**Table:**

	*U*^11^	*U*^22^	*U*^33^	*U*^12^	*U*^13^	*U*^23^
B1	0.0358 (9)	0.0501 (11)	0.0424 (10)	−0.0072 (8)	−0.0031 (8)	0.0077 (8)
O1	0.0447 (7)	0.0665 (8)	0.0345 (6)	0.0031 (6)	0.0016 (5)	0.0079 (5)
N1	0.0361 (7)	0.0375 (7)	0.0315 (7)	−0.0030 (5)	0.0009 (5)	0.0049 (5)
N2	0.0397 (7)	0.0423 (8)	0.0406 (7)	0.0009 (6)	−0.0056 (6)	−0.0007 (6)
C1	0.0324 (7)	0.0329 (7)	0.0354 (8)	−0.0015 (6)	−0.0026 (6)	−0.0015 (6)
C2	0.0303 (7)	0.0369 (8)	0.0375 (8)	0.0020 (6)	−0.0049 (6)	0.0006 (6)
C3	0.0290 (7)	0.0423 (8)	0.0386 (8)	0.0001 (6)	0.0019 (6)	−0.0028 (6)
C4	0.0390 (8)	0.0407 (8)	0.0298 (7)	0.0001 (6)	−0.0022 (6)	−0.0017 (6)
C5	0.0335 (8)	0.0454 (9)	0.0370 (8)	0.0026 (6)	−0.0075 (6)	0.0008 (7)
C6	0.0273 (7)	0.0431 (8)	0.0387 (8)	−0.0005 (6)	0.0005 (6)	−0.0017 (6)
C7	0.0574 (12)	0.0904 (15)	0.0391 (10)	0.0067 (10)	0.0129 (8)	0.0114 (10)
C8	0.0417 (8)	0.0327 (8)	0.0407 (8)	−0.0009 (6)	−0.0021 (7)	0.0070 (6)
C9	0.0418 (8)	0.0314 (7)	0.0385 (8)	−0.0039 (6)	−0.0003 (7)	0.0005 (6)
C10	0.0337 (7)	0.0379 (8)	0.0277 (7)	0.0013 (6)	0.0039 (6)	−0.0001 (6)
C11	0.0435 (8)	0.0324 (8)	0.0336 (8)	0.0030 (6)	0.0000 (6)	−0.0006 (6)
C12	0.0434 (8)	0.0334 (8)	0.0328 (8)	−0.0032 (6)	0.0018 (6)	0.0000 (6)
C13	0.0459 (9)	0.0540 (10)	0.0507 (10)	−0.0061 (8)	−0.0117 (8)	−0.0059 (8)
C14	0.0568 (12)	0.0560 (11)	0.0618 (12)	0.0110 (9)	−0.0197 (9)	0.0004 (9)
B1'	0.0333 (9)	0.0635 (12)	0.0392 (10)	−0.0031 (8)	0.0021 (8)	0.0000 (8)
O1'	0.0467 (7)	0.0583 (7)	0.0345 (6)	0.0036 (5)	−0.0030 (5)	−0.0021 (5)
N1'	0.0349 (7)	0.0465 (8)	0.0315 (7)	−0.0003 (5)	0.0042 (5)	−0.0018 (5)
N2'	0.0345 (7)	0.0418 (7)	0.0378 (7)	−0.0006 (5)	−0.0013 (5)	0.0025 (5)
C1'	0.0328 (8)	0.0339 (7)	0.0393 (8)	−0.0006 (6)	−0.0021 (6)	0.0000 (6)
C2'	0.0308 (8)	0.0505 (9)	0.0358 (8)	−0.0013 (6)	−0.0052 (6)	−0.0034 (7)
C3'	0.0300 (8)	0.0469 (9)	0.0403 (9)	−0.0005 (6)	0.0006 (6)	−0.0014 (7)
C4'	0.0398 (8)	0.0323 (7)	0.0345 (8)	0.0018 (6)	−0.0025 (6)	−0.0024 (6)
C5'	0.0351 (8)	0.0343 (8)	0.0431 (9)	0.0009 (6)	−0.0108 (7)	−0.0014 (6)
C6'	0.0291 (7)	0.0354 (8)	0.0449 (9)	0.0008 (6)	−0.0013 (6)	−0.0001 (6)
C7'	0.0538 (11)	0.0577 (11)	0.0399 (9)	0.0058 (8)	0.0071 (8)	−0.0059 (8)
C8'	0.0408 (8)	0.0427 (8)	0.0333 (8)	0.0051 (7)	0.0034 (6)	0.0034 (6)
C9'	0.0402 (8)	0.0365 (8)	0.0345 (8)	−0.0002 (6)	0.0049 (6)	0.0004 (6)
C10'	0.0329 (7)	0.0383 (8)	0.0292 (7)	0.0024 (6)	0.0064 (6)	−0.0022 (6)
C11'	0.0375 (8)	0.0358 (8)	0.0346 (8)	0.0037 (6)	0.0041 (6)	0.0007 (6)
C12'	0.0386 (8)	0.0375 (8)	0.0354 (8)	−0.0011 (6)	0.0067 (6)	−0.0026 (6)
C13'	0.0406 (9)	0.0491 (9)	0.0454 (9)	−0.0092 (7)	0.0018 (7)	−0.0001 (7)
C14'	0.0403 (9)	0.0520 (10)	0.0455 (10)	0.0028 (7)	−0.0049 (7)	0.0069 (7)

### Geometric parameters (Å, º)

**Table:**

B1—N1	1.597 (2)	B1'—N1'	1.595 (2)
B1—C1	1.608 (2)	B1'—C1'	1.611 (2)
B1—H1B	1.13 (2)	B1'—H1'B	1.150 (19)
B1—H2B	1.14 (2)	B1'—H2'B	1.166 (19)
O1—C4	1.3752 (19)	O1'—C4'	1.370 (2)
O1—C7	1.413 (2)	O1'—C7'	1.423 (2)
N1—C12	1.342 (2)	N1'—C12'	1.347 (2)
N1—C8	1.350 (2)	N1'—C8'	1.350 (2)
N2—C10	1.338 (2)	N2'—C10'	1.344 (2)
N2—C14	1.455 (2)	N2'—C14'	1.456 (2)
N2—C13	1.459 (2)	N2'—C13'	1.458 (2)
C1—C2	1.396 (2)	C1'—C2'	1.390 (2)
C1—C6	1.402 (2)	C1'—C6'	1.400 (2)
C2—C3	1.389 (2)	C2'—C3'	1.397 (2)
C2—H2A	0.9500	C2'—H2'A	0.9500
C3—C4	1.387 (2)	C3'—C4'	1.380 (2)
C3—H3A	0.9500	C3'—H3'A	0.9500
C4—C5	1.388 (2)	C4'—C5'	1.392 (2)
C5—C6	1.381 (2)	C5'—C6'	1.383 (2)
C5—H5A	0.9500	C5'—H5'A	0.9500
C6—H6A	0.9500	C6'—H6'A	0.9500
C7—H7A	0.996 (14)	C7'—H7'1	0.996 (14)
C7—H7B	0.996 (14)	C7'—H7'2	0.996 (14)
C7—H7C	0.996 (14)	C7'—H7'3	0.996 (14)
C8—C9	1.361 (2)	C8'—C9'	1.364 (2)
C8—H8A	0.9500	C8'—H8'A	0.9500
C9—C10	1.419 (2)	C9'—C10'	1.419 (2)
C9—H9A	0.9500	C9'—H9'A	0.9500
C10—C11	1.413 (2)	C10'—C11'	1.418 (2)
C11—C12	1.366 (2)	C11'—C12'	1.364 (2)
C11—H11A	0.9500	C11'—H11B	0.9500
C12—H12A	0.9500	C12'—H12B	0.9500
C13—H13A	0.967 (13)	C13'—H13D	0.955 (12)
C13—H13B	0.967 (13)	C13'—H13E	0.955 (12)
C13—H13C	0.967 (13)	C13'—H13F	0.955 (12)
C14—H14A	0.973 (15)	C14'—H14D	0.985 (12)
C14—H14B	0.973 (15)	C14'—H14E	0.985 (12)
C14—H14C	0.973 (15)	C14'—H14F	0.985 (12)
			
N1—B1—C1	110.83 (13)	N1'—B1'—C1'	110.96 (13)
N1—B1—H1B	108.2 (10)	N1'—B1'—H1'B	107.0 (10)
C1—B1—H1B	109.7 (10)	C1'—B1'—H1'B	110.9 (10)
N1—B1—H2B	105.5 (10)	N1'—B1'—H2'B	103.9 (10)
C1—B1—H2B	112.2 (11)	C1'—B1'—H2'B	114.0 (10)
H1B—B1—H2B	110.2 (14)	H1'B—B1'—H2'B	109.7 (14)
C4—O1—C7	117.30 (14)	C4'—O1'—C7'	117.41 (13)
C12—N1—C8	116.49 (13)	C12'—N1'—C8'	116.54 (13)
C12—N1—B1	122.47 (13)	C12'—N1'—B1'	122.52 (14)
C8—N1—B1	121.02 (13)	C8'—N1'—B1'	120.93 (14)
C10—N2—C14	121.03 (14)	C10'—N2'—C14'	120.93 (13)
C10—N2—C13	121.04 (13)	C10'—N2'—C13'	122.19 (13)
C14—N2—C13	117.81 (14)	C14'—N2'—C13'	116.18 (14)
C2—C1—C6	115.29 (14)	C2'—C1'—C6'	115.03 (14)
C2—C1—B1	125.10 (13)	C2'—C1'—B1'	125.57 (14)
C6—C1—B1	119.59 (14)	C6'—C1'—B1'	119.39 (14)
C3—C2—C1	123.11 (14)	C1'—C2'—C3'	123.40 (15)
C3—C2—H2A	118.4	C1'—C2'—H2'A	118.3
C1—C2—H2A	118.4	C3'—C2'—H2'A	118.3
C4—C3—C2	119.50 (14)	C4'—C3'—C2'	119.48 (15)
C4—C3—H3A	120.2	C4'—C3'—H3'A	120.3
C2—C3—H3A	120.2	C2'—C3'—H3'A	120.3
O1—C4—C3	124.62 (14)	O1'—C4'—C3'	125.02 (15)
O1—C4—C5	116.14 (14)	O1'—C4'—C5'	115.90 (14)
C3—C4—C5	119.23 (14)	C3'—C4'—C5'	119.06 (15)
C6—C5—C4	120.01 (14)	C6'—C5'—C4'	120.00 (14)
C6—C5—H5A	120.0	C6'—C5'—H5'A	120.0
C4—C5—H5A	120.0	C4'—C5'—H5'A	120.0
C5—C6—C1	122.85 (14)	C5'—C6'—C1'	123.02 (14)
C5—C6—H6A	118.6	C5'—C6'—H6'A	118.5
C1—C6—H6A	118.6	C1'—C6'—H6'A	118.5
O1—C7—H7A	109.5	O1'—C7'—H7'1	109.5
O1—C7—H7B	109.5	O1'—C7'—H7'2	109.5
H7A—C7—H7B	109.5	H7'1—C7'—H7'2	109.5
O1—C7—H7C	109.5	O1'—C7'—H7'3	109.5
H7A—C7—H7C	109.5	H7'1—C7'—H7'3	109.5
H7B—C7—H7C	109.5	H7'2—C7'—H7'3	109.5
N1—C8—C9	123.92 (14)	N1'—C8'—C9'	123.76 (14)
N1—C8—H8A	118.0	N1'—C8'—H8'A	118.1
C9—C8—H8A	118.0	C9'—C8'—H8'A	118.1
C8—C9—C10	120.23 (14)	C8'—C9'—C10'	120.40 (14)
C8—C9—H9A	119.9	C8'—C9'—H9'A	119.8
C10—C9—H9A	119.9	C10'—C9'—H9'A	119.8
N2—C10—C11	122.89 (14)	N2'—C10'—C9'	123.00 (14)
N2—C10—C9	121.99 (14)	N2'—C10'—C11'	122.00 (14)
C11—C10—C9	115.11 (14)	C9'—C10'—C11'	114.99 (14)
C12—C11—C10	120.41 (14)	C12'—C11'—C10'	120.44 (14)
C12—C11—H11A	119.8	C12'—C11'—H11B	119.8
C10—C11—H11A	119.8	C10'—C11'—H11B	119.8
N1—C12—C11	123.83 (14)	N1'—C12'—C11'	123.83 (14)
N1—C12—H12A	118.1	N1'—C12'—H12B	118.1
C11—C12—H12A	118.1	C11'—C12'—H12B	118.1
N2—C13—H13A	109.5	N2'—C13'—H13D	109.5
N2—C13—H13B	109.5	N2'—C13'—H13E	109.5
H13A—C13—H13B	109.5	H13D—C13'—H13E	109.5
N2—C13—H13C	109.5	N2'—C13'—H13F	109.5
H13A—C13—H13C	109.5	H13D—C13'—H13F	109.5
H13B—C13—H13C	109.5	H13E—C13'—H13F	109.5
N2—C14—H14A	109.5	N2'—C14'—H14D	109.5
N2—C14—H14B	109.5	N2'—C14'—H14E	109.5
H14A—C14—H14B	109.5	H14D—C14'—H14E	109.5
N2—C14—H14C	109.5	N2'—C14'—H14F	109.5
H14A—C14—H14C	109.5	H14D—C14'—H14F	109.5
H14B—C14—H14C	109.5	H14E—C14'—H14F	109.5
			
C1—B1—N1—C12	117.49 (16)	C1'—B1'—N1'—C12'	106.68 (17)
C1—B1—N1—C8	−63.9 (2)	C1'—B1'—N1'—C8'	−72.92 (19)
N1—B1—C1—C2	−21.1 (2)	N1'—B1'—C1'—C2'	−3.2 (2)
N1—B1—C1—C6	160.91 (14)	N1'—B1'—C1'—C6'	177.29 (14)
C6—C1—C2—C3	−0.4 (2)	C6'—C1'—C2'—C3'	−0.2 (2)
B1—C1—C2—C3	−178.43 (15)	B1'—C1'—C2'—C3'	−179.71 (16)
C1—C2—C3—C4	−0.5 (2)	C1'—C2'—C3'—C4'	−0.6 (3)
C7—O1—C4—C3	−7.4 (2)	C7'—O1'—C4'—C3'	−7.1 (2)
C7—O1—C4—C5	173.96 (17)	C7'—O1'—C4'—C5'	174.38 (14)
C2—C3—C4—O1	−177.46 (15)	C2'—C3'—C4'—O1'	−177.51 (15)
C2—C3—C4—C5	1.1 (2)	C2'—C3'—C4'—C5'	1.0 (2)
O1—C4—C5—C6	177.77 (14)	O1'—C4'—C5'—C6'	178.10 (13)
C3—C4—C5—C6	−0.9 (2)	C3'—C4'—C5'—C6'	−0.5 (2)
C4—C5—C6—C1	0.1 (2)	C4'—C5'—C6'—C1'	−0.3 (2)
C2—C1—C6—C5	0.6 (2)	C2'—C1'—C6'—C5'	0.6 (2)
B1—C1—C6—C5	178.75 (15)	B1'—C1'—C6'—C5'	−179.77 (15)
C12—N1—C8—C9	0.4 (2)	C12'—N1'—C8'—C9'	0.6 (2)
B1—N1—C8—C9	−178.28 (15)	B1'—N1'—C8'—C9'	−179.78 (14)
N1—C8—C9—C10	0.2 (2)	N1'—C8'—C9'—C10'	0.9 (2)
C14—N2—C10—C11	−1.7 (2)	C14'—N2'—C10'—C9'	174.39 (14)
C13—N2—C10—C11	−177.60 (15)	C13'—N2'—C10'—C9'	4.4 (2)
C14—N2—C10—C9	179.35 (16)	C14'—N2'—C10'—C11'	−6.6 (2)
C13—N2—C10—C9	3.4 (2)	C13'—N2'—C10'—C11'	−176.65 (14)
C8—C9—C10—N2	178.37 (14)	C8'—C9'—C10'—N2'	177.12 (14)
C8—C9—C10—C11	−0.7 (2)	C8'—C9'—C10'—C11'	−1.9 (2)
N2—C10—C11—C12	−178.48 (14)	N2'—C10'—C11'—C12'	−177.51 (14)
C9—C10—C11—C12	0.6 (2)	C9'—C10'—C11'—C12'	1.5 (2)
C8—N1—C12—C11	−0.5 (2)	C8'—N1'—C12'—C11'	−1.0 (2)
B1—N1—C12—C11	178.13 (15)	B1'—N1'—C12'—C11'	179.36 (14)
C10—C11—C12—N1	0.1 (2)	C10'—C11'—C12'—N1'	−0.1 (2)

### Hydrogen-bond geometry (Å, º)

Cg is the centroid of the C1'–C6' ring.

**Table:**

*D*—H···*A*	*D*—H	H···*A*	*D*···*A*	*D*—H···*A*
C9—H9*A*···*Cg*	0.95	3.12	4.069 (2)	178
C13—H13*A*···*Cg*	0.97	3.12	3.662 (2)	112
C13—H13*C*···*Cg*	0.97	3.23	3.662 (2)	109
